# UDCA ameliorates inflammation driven EMT by inducing TGR5 dependent SOCS1 expression in mouse macrophages

**DOI:** 10.1038/s41598-024-75516-9

**Published:** 2024-10-16

**Authors:** Ashna Fathima, Trinath Jamma

**Affiliations:** Cell Signaling Laboratory, Department of Biological Sciences, Birla Institute of Technology, and Science-Pilani Hyderabad Campus, Jawahar Nagar, Shameerpet Mandal, Hyderabad, 500078 Telangana India

**Keywords:** Intestinal inflammation, Colitis-associated cancer, Secondary bile acids, Cytokines, SOCS1, Cancer, Cell biology, Immunology

## Abstract

Long-standing chronic inflammation of the digestive tract leads to Inflammatory Bowel Diseases (IBD), comprising Crohn’s Disease (CD) and Ulcerative colitis (UC). The persistent prevalence of these conditions in the gut is a predisposing factor for Colitis-Associated Cancer (CAC), one of the most common sub-types of Colorectal Cancer (CRC), emphasizing the role of inflammation in tumorigenesis. Therefore, targeted intervention of chronic intestinal inflammation is a potential strategy for preclusion and treatment of inflammation-driven malignancies. The association between bile acids (BA) and gut immune homeostasis has been explored in the recent past. However, the exact downstream mechanism by which secondary BA successfully regulating intestinal inflammation and inflammation-dependent CAC is unclear. Our study demonstrated that Ursodeoxycholic acid (UDCA), a secondary bile acid of host gut microbial origin, finetunes the dialogue between activated macrophages and intestinal epithelial cells, modulating inflammation-driven epithelial-mesenchymal transition (EMT), a hallmark of cancer. UDCA treatment and dependency on the TGR5/GPBAR1 receptor significantly upregulated the Suppressor of Cytokine Signaling 1 (SOCS1) expression, contributing to the regulation of pro-inflammatory cytokines in activated macrophages. In this study, we also noticed heightened expression of SOCS1 in UDCA-mitigated CAC in the AOM-DSS mouse model with reduced inflammatory gene expression. Overall, our observations highlight the possible utility of UDCA for inflammation-driven intestinal cancer.

**Supplementary Information:**

The online version contains supplementary material available at 10.1038/s41598-024-75516-9.

## Introduction

One of the largest interfaces between the host and the luminal antigens of exogenous origin in the human body is the gastrointestinal (GI) tract, colonized with commensal bacteria, archaea, and eukarya, collectively called the gut microbiota^1^. The gut microbiota plays a prominent role not only in food digestion, absorption, and energy harvest but also in the host’s neurological functions and signaling, regulation of metabolism, maintaining gut integrity, and tailoring host immunity, collectively contributing to gut immune homeostasis^2,3^. The altered gut microbial composition can arise due to changes in dietary habits, lifestyle, and stress often associated with the dysregulation of physiological and metabolic processes^4^. Dysbiosis has been implicated in the pathogenesis of many gastrointestinal inflammatory diseases, such as inflammatory bowel diseases (IBD) and functional dyspepsia^5^.

Modulatory mechanisms of gut microbiota in the development of metabolic diseases have been studied in the recent past. The host-gut microbial crosstalk via the synthesis of a myriad of metabolites has gained interest, as these metabolites possess potential bioactivity ranging from influencing metabolic responses to regulation of inflammation and maintenance of gut epithelial cell integrity^3,6^. The importance of short-chain fatty acids (SCFA), branched-chain amino acids, or choline metabolism has been well-reported^6^. Bile acid (BA) conversion is another essential metabolic feature of the gut microbiota with a substantial impact on host metabolism, and the therapeutic potential of intervening with BA-dependent pathways is highly appreciated and exploited to develop novel therapeutics^7^. Clinical survey shows that IBD patients experience a loss of microbial diversity, contributing to a reduction in the reabsorption of BA and variation in the rate of intestinal BA metabolism^8^. This observation is also supported by metabolomic studies, which reveal a consistent defect in BA metabolism with an increase in primary BAs and a reduction in secondary BAs in IBD patients^9^.

BA are known ligands for members of the Nuclear Receptor (NR) superfamily: the Farnesoid X Receptor (FXR, NR1H4), the Pregnane X Receptor (PXR, NR1H2), and the Vitamin D Receptor (VDR, NR1H1). Secondary BA also activate G-protein-coupled receptors (GPCRs), including TGR5 (also known as M-BAR, GPBAR1, or BG37)^10^. TGR5 and FXR are highly expressed by intestinal epithelial cells^11^, non-epithelial cells, including neurons^12,13^, and vascular and liver endothelial cells^14,15^. Furthermore, both receptors are also highly represented in immune cells, monocytes and macrophages, dendritic cells (DCs), natural killer (NK), innate lymphoid cells(ILC), and NKT cells^16^. Activation of BARs in macrophages, DCs, and NKT results in several regulatory functions and contributes to maintaining the tolerogenic state of the intestinal innate immunity in response to constant exposure to dietary xenobiotics and antigens in the gut^11,16^.

One of the characteristics of IBD is defective gut epithelial barrier function in combination with immune dysregulation, and this leads to intestinal barrier breach by various bacterial products activating Toll-like receptors (TLR) and nucleotide-binding and oligomerization domain (NOD)-like receptor (NLR) pathways^17^. Bacterial LPS is an active agent that interacts with host cells, such as monocytes and macrophages and triggers the pathogenesis of endotoxin shock. This leads to the production of inflammatory mediators such as cytokines, including IL-1β, IL-6, TNF-α, IL-12, and IFNγ^18^. Cytokines are vital players in intestinal homeostasis and are also associated with the pathology of Crohn’s disease (CD) and Ulcerative colitis (UC), two forms of IBD^19,20^. BA signaling is known to suppress the pro-inflammatory phenotype of intestinal cells by the reduced release of TNF-α, IL-1β, IL-6, or IL-12^21^. Studies have also reported that BA stimulates the production of anti-inflammatory cytokines, promoting epithelial barrier renewal^22^. The importance of secondary BAs in regulating inflammation and inflammation-driven malignancies and understanding the therapeutic potential of these secondary metabolites remain the primary objectives of our research.

A homeostatic immune system in a healthy state is an outcome of functional cooperation between positive and negative regulators of immune cell signaling pathways. Therefore, potent negative regulators are required to reduce the inflammatory signature to maintain homeostasis. One of the negative regulators of inflammation includes the Suppressor of Cytokine Signaling (SOCS)^23^. Blocking the release of cytokines or modulating the response to the inflammatory cytokines by upregulating the expression of these negative regulators may provide a mechanism to modulate inflammatory disorders.

Here, we identified a secondary BA that intercepts the activated macrophage-driven epithelial-to-mesenchymal transition (EMT) in the mouse intestinal epithelial cell line, CT26. Ursodeoxycholic acid (UDCA) is a naturally occurring secondary BA derived from the gut microbial metabolism of chenodeoxycholic acid (CDCA)^24^. In a healthy human gut, concentrations of secondary BA fall in the hundreds of micromolar range, potentiating UDCA as a safe therapeutic option with fewer side effects even at a relatively higher concentration (> 100µM)^24,25^. While its mechanisms of action are not well defined, it is believed that the therapeutic properties of UDCA are primarily due to its anti-inflammatory and cytoprotective actions^25^. We observed that UDCA ameliorated the production of pro-inflammatory cytokines in LPS-stimulated macrophages and independently upregulated the expression of SOCS1. UDCA significantly impacted the activated macrophage-driven EMT in CT26 cells cultured in activated macrophage-conditioned media. siRNA-mediated silencing of SOCS1 and TGR5 receptor in macrophages (RAW264.7 cells) drastically reversed the immuno-modulatory effect of UDCA. Further, dietary supplementation of mice with UDCA reduced the incidence of tumors compared to the control diet in the AOM-DSS mouse model of inflammation-driven colon cancer. Altogether, these results suggest a potential therapeutic role for UDCA in mitigating inflammation-driven CAC with a possible involvement of SOCS1 in vitro and in vivo.

## Results

### UDCA regulates activated macrophage-driven EMT of intestinal epithelial cells (IECs)

The bidirectional communication between the intestinal epithelium and the immune system is convolutedly controlled in health and disease^18^. Upon exposure to external stimuli, these immune components can modulate biphasic epithelial permeability^26^. The gut lining is in constant exposure to xenobiotics, imposing a challenge in maintaining intestinal barrier integrity^18,26^. Loss of barrier function leads to an influx of luminal pathogens and a subsequent aberrant immune response, which causes tissue damage and can trigger colitis-associated cancers^27^. Our study focused on understanding the regulatory mechanism of BA in modulating inflammation-driven malignancies.

**Fig. 1:**
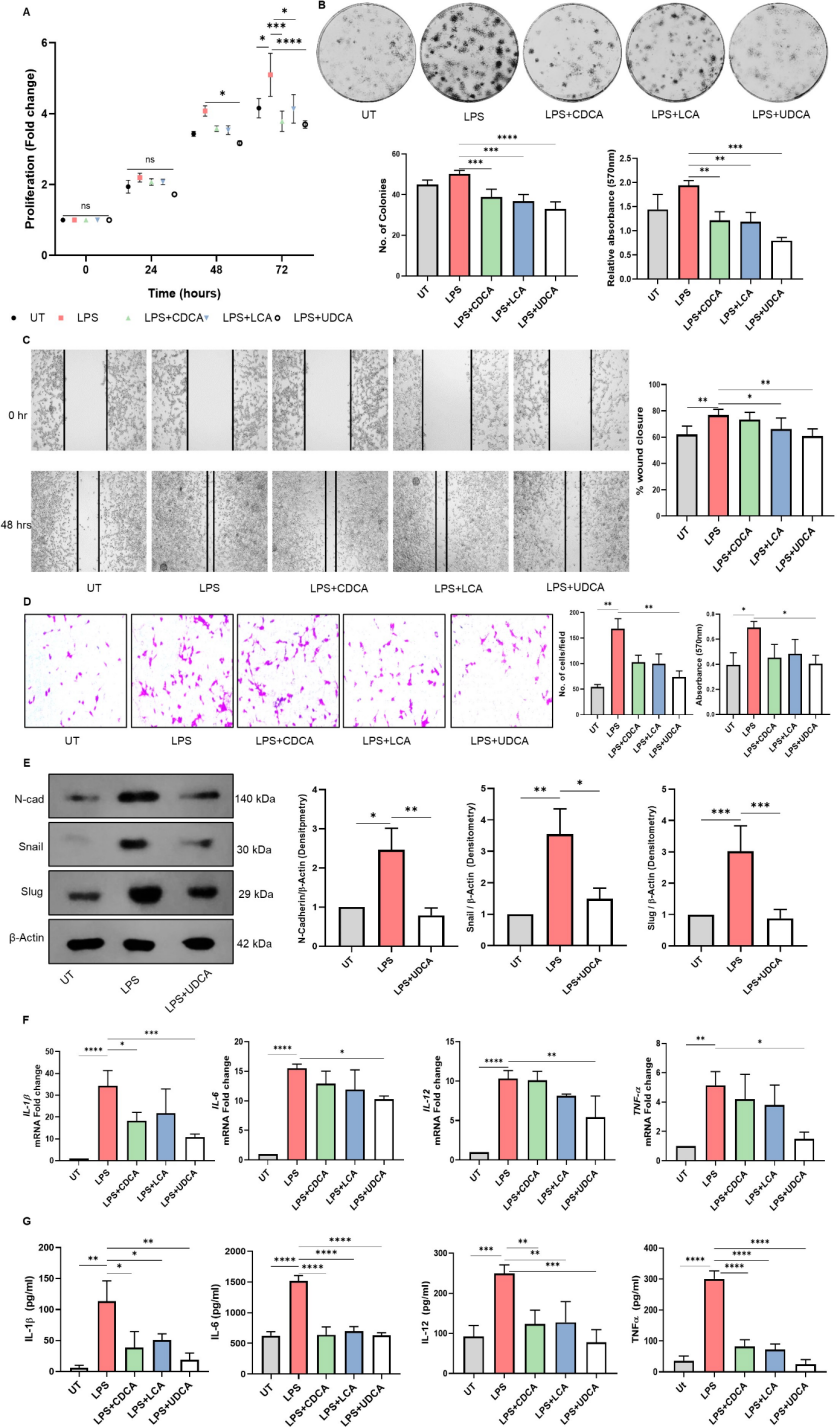
Impact of BAs on activated macrophage-driven EMT of IECs. Murine peritoneal macrophages were treated with 100µM Bile acids of interest and activated by 100ng/ml of LPS. After 24 h of incubation, the culture supernatant was collected. CT26 cells were treated with macrophage conditioned medium. **(A)** MTT assay was performed to analyze the cell proliferation fold change of macrophage culture medium-treated CT26 cells. **(B)** Impact on the colony formation ability of supernatant-treated CT26 cells upon BA treatment of macrophages showed that UDCA had the most regulatory effect among all the tested BAs. Quantified histogram representation of relative colony number and the absorbance of stain taken up by the cells. **(C)** Representative images from in vitro wound healing assays demonstrate that at 48 h, the cell migration of macrophage-conditioned medium-treated CT26 cells was significantly reduced upon UDCA treatment. **(D)** Representative images showing the results of Transwell invasion assay of CT26 cells treated with macrophage-conditioned medium. Quantified histogram representation of the relative number of cells per field and the absorbance of stain taken up by the cells. **(E)** Post-translational expression of EMT markers N-Cadherin, Snail, and Slug was analyzed by western blotting. Histograms representing densitometric analysis w.r.t β-Actin. **F**,** G)** Pro-inflammatory cytokine expression and secretion by BA-treated activated macrophages were quantified by qPCR and ELISA, respectively. Data represented as mean ± SD of at least triplicate experiments. *P < 0.05, **P < 0.01, ***P < 0.001. Raw images of western blots are provided as supplementary file.

Firstly, macrophages were activated by LPS in the presence of BAs for 24 h. The supernatant was collected for further experiments. The crosstalk between activated macrophage and the ability of BAs to influence inflammation-driven EMT was analyzed by performing a series of experiments. We first investigated the impact of BAs on CT26 proliferation by performing MTT assay. The supernatant from activated macrophages was brought in contact with CT26 cells. It was observed that all the tested BAs had a significant effect on cell proliferation by 72 h of incubation. Meanwhile, UDCA exhibited a marked reduction in the LPS-activated macrophage-induced proliferation at 48 h of incubation and thereafter as well **(**Fig. 1A). We further investigated the impact of these BAs on regulating cancerous phenotype by performing colony-forming, wound healing as well as invasion assays. As expected, cells exposed to LPS-activated macrophage-conditioned media had more profound colonies than those in the control group. Upon BA treatment, we could observe a marked reduction in the number of colonies, thus reversing the effect of LPS-activated macrophages (Fig. 1B). A similar impact was observed in the epithelial cells’ migration and invasion ability (Fig. 1C, D). Based on these experiments, it was inferred that UDCA imparted the most significant impact on regulating inflammatory stimuli-induced changes in intestinal epithelial cells.

EMT is a well-described pathogenetic event in cancer progression and metastasis. The EMT process is tightly connected to inflammation, recently identified as a key inducer of metastasis during cancer progression^28^. To investigate whether UDCA-regulated cell migration and proliferation are associated with EMT, we performed a western blot analysis of mesenchymal markers in conditioned media-treated CT26 cells. We found that the heightened expression of mesenchymal markers N-CADHERIN and transcription factors, such as SNAIL and SLUG, induced by activated macrophage was brought down by UDCA (Fig. 1E). The impact BAs on LPS-induced pro-inflammatory cytokine production in macrophages has been highlighted in prior studies^21,29^. Confirming this, we also observed that UDCA treatment led to a concomitant reduction in the expression and secretion of pro-inflammatory cytokines in LPS-primed macrophages (Fig. 1F, G). Together, these results demonstrated that exposure to secondary BA, UDCA, could regulate the crosstalk between activated macrophages and epithelial cells.

### TGR5 facilitates UDCA-mediated regulation of activated macrophage-driven EMT

**Fig. 2:**
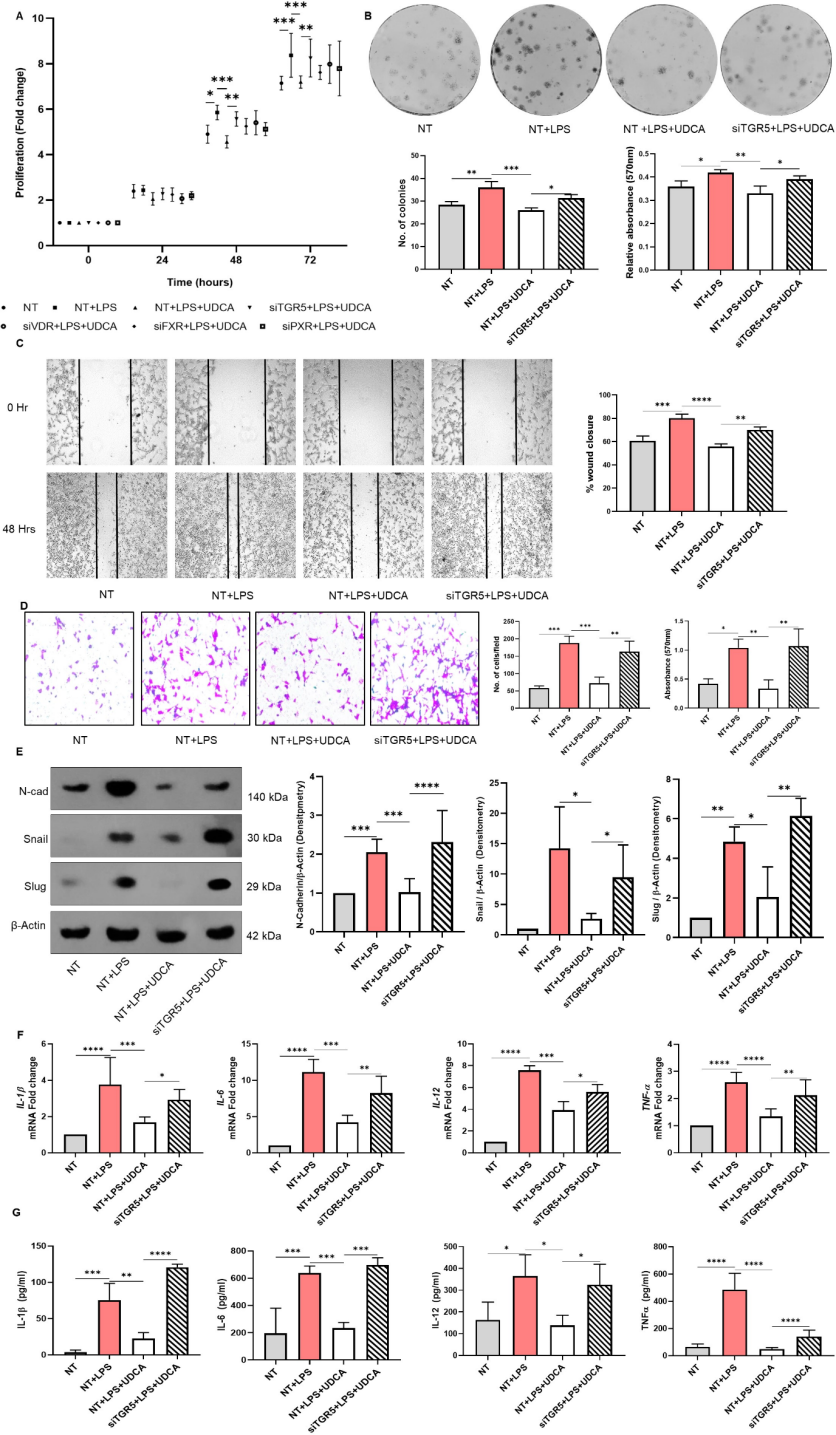
Impact of BAR silencing on activated macrophage-driven EMT of IECs. RAW264.7 Macrophages were transfected with BAR-specific siRNAs. After 6 h of incubation followed by 24-hour rest, the cells were treated with 100µM UDCA and activated by 100ng/ml of LPS. After 24 h of incubation, the culture supernatant was collected. CT26 cells were treated with macrophage conditioned medium. **(A)** MTT assay was performed to analyze the effect of BA and BAR silencing on supernatant-treated CT26 cells proliferation. **(B)** Impact was observed on the colony formation of CT26 cells upon TGR5 silencing. Quantified histogram representation of relative colony number and the absorbance of stain taken up by the cells. **(C)** Representative images from in vitro wound healing assays after 48 h of incubation demonstrated that cell migration significantly reduced on UDCA treatment but was reversed upon TGR5 silencing. **(D)** Representative images showing the results of transwell invasion assay of CT26 cells treated with TGR5 deficient macrophage-conditioned medium. Quantified histogram representation of the relative number of cells per field and the absorbance of stain taken up by the cells. **(E)** Western blot analysis of protein level expression of N-Cadherin, Snail, and Slug in TGR5 deficient-macrophage conditioned media-treated CT26 cells after 48 h of incubation. Densitometry w.r.t β-Actin represented as mean ± SD of triplicate experiments. **F**,** G)** Pro-inflammatory cytokine expression and secretion by BA-treated and TGR5 deficient macrophages were quantified by qPCR and ELISA, respectively. Data represented as mean ± SD of at least triplicate experiments. *P < 0.05, **P < 0.01, ***P < 0.001, ****P < 0.000. Raw images of western blots are provided as supplementary files.

Many BAs are shown to modulate multiple physiological and pathological processes via a family of receptors known as bile acid-activated receptors or bile acid receptors (BAR)^10^. Deciphering the precise signaling pathway triggered by BA and its cognate receptor regulating the inflammatory response in activated macrophages remains the primary objective of our study. To identify the BAR facilitating UDCA signaling, siRNA-mediated knockdown of BARs such as TGR5, FXR, VDR, and PXR was performed in macrophages. The BAR siRNA transfected cells were subjected to LPS activation in combination with UDCA treatment. CT26 cells were cultured in the thus obtained conditioned medium, and we could observe that UDCA-mediated inhibition of cell proliferation was markedly hampered upon TGR5 silencing as compared to the rest of the receptors (Fig. 2A). To establish further the role of TGR5 in UDCA signaling, colony-forming assay, cell migration assay, and invasion assay were performed. The reduction in the proliferation and migration of CT26 upon treatment with the UDCA-conditioned medium was restored considerably when the TGR5 receptor was silenced in the macrophages (Fig. 2B-D).

We further evaluated the expression of mesenchymal markers. We found a similar outcome: the post-translational expression of N-CADHERIN, SNAIL, and SLUG was upregulated upon TGR5 silencing **(**Fig. 2E). Studies have reported that TGR5 deficiency in macrophages was coupled with increased cytokine secretion and macrophage migration^30^. On par with this, we observed that the inhibitory effect of UDCA on the mRNA expression and the secretion of pro-inflammatory cytokines IL-1β, IL-6, IL-12, and TNF-α were reversed in the absence of TGR5 receptor (Fig. 2F, G). Together, our results highlight the significance of the TGR5 receptor in expediating UDCA-induced downregulation of activated macrophage-driven EMT.

### UDCA induces SOCS1 expression via TGR5

SOCS proteins are known to participate in multiple cellular functions, including the regulation of pro-inflammatory cytokine signaling, and their expression is regulated by several mechanisms. SOCS1 expression is predominantly regulated in cancer cells, indicating its potential role in cancer progression^31^. The antitumor potential of SOCS1 has been explored in the recent past. Studies have shown that SOCS1-deficient mice are more susceptible to autoinflammatory and autoimmune diseases^32,33^. SOCS1 gene methylation has been well correlated with hepatocellular health as it is known to play a significant role in liver inflammation, hepatocellular cancer, and several other cancers, including colorectal cancer^34–36^. These findings suggest the potential of SOCS1 in modulating inflammation-associated cancer.

Clinical implications of SOCS isoform expression in Colon Adenocarcinoma (COAD) patients were analyzed using a publicly available database, OncoDB (*oncodb.org*). Around 430 COAD patients (TCGA) were grouped into categories based on the pathological stages. The expression of SOCS1, SOCS2, SOCS3, and CISH was analyzed. Among the isoforms, SOCS1 exhibited an interesting expression pattern. No significant change was observed in SOCS1 gene expression among Normal and COAD participants. However, upon further analysis of SOCS1 expression at different pathological stages, there was an increase in SOCS1 expression in the initial stages of tumor progression. In contrast, there was a significant reduction as the severity of the disease increased (Supplementary Fig**. ****S1****)**. This observation may suggest that upregulating the expression of SOCS1 could be a potential mechanism to regulate disease progression.

**Fig. 3:**
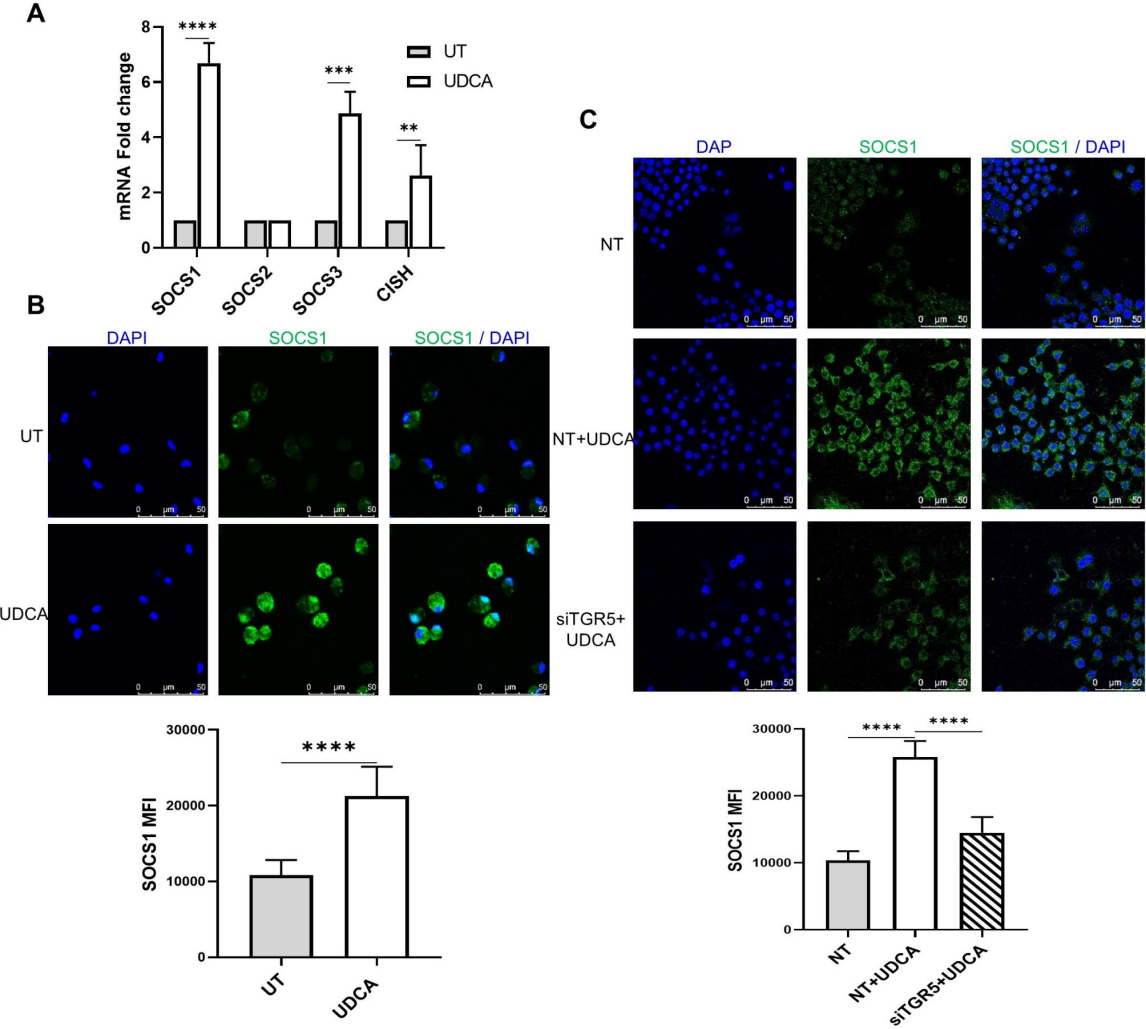
UDCA upregulates SOCS1 expression via the TGR5 receptor.**(A)** mRNA level expression of SOCS isoforms by qPCR showed a significant increase in the expression of SOCS1 in UDCA-treated mice peritoneal macrophages. **(B)** The expression of SOCS1 proteins in UDCA-treated mice peritoneal macrophages was analyzed by Immunofluorescence staining and detection at 63X objective magnification. The mean fluorescence intensity (MFI) of the individual cells per field imaged was quantified. **(C)** The expression of SOCS1 proteins in TGR5 deficit RAW264.7 macrophages was analyzed by Immunofluorescence staining and detection at 63X objective magnification. The mean fluorescence intensity (MFI) of the individual cells per field imaged was quantified. Data represented as mean ± SD of at least triplicate experiments. **P < 0.01, ***P < 0.001, ****P < 0.0001.

To address this, mouse macrophages were examined in this study to assess the potential of UDCA in regulating the expression of SOCS isoforms. As shown **(**Fig. 3A), mRNA expression of SOCS1 in murine macrophages was upregulated in the presence of UDCA. The protein expression was also analyzed by immunofluorescence. In concordance with the real-time PCR data, SOCS1 protein expression was found to be significantly increased when the macrophages were treated with UDCA (Fig. 3B). To validate the role of TGR5 in UDCA-induced expression of SOCS1, siRNA-mediated transient knockdown of TGR5 receptor was carried out. Reduction in the expression of TGR5 in macrophages is confirmed by quantitative real-time PCR (Supplementary Fig. S2A)**.** We observed a significant reduction in the expression of SOCS1 in TGR5 deficient RAW264.7 macrophages (Fig. 3C). The data suggested that UDCA induced the expression of SOCS1 via TGR5.

### siRNA-mediated silencing of SOCS1 in macrophages interferes with the ameliorating effect of UDCA on activated macrophage-driven EMT

**Fig. 4:**
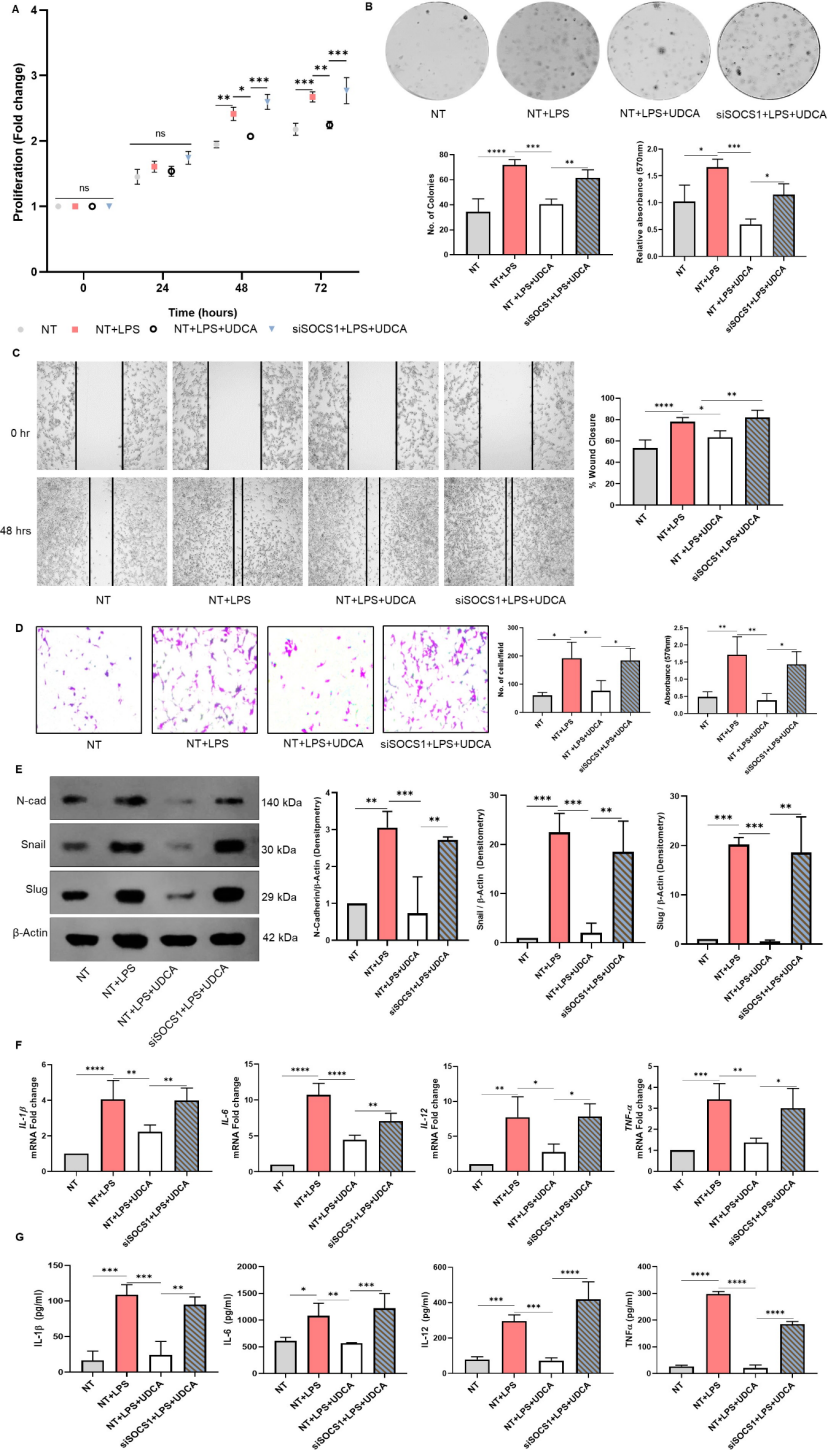
Impact of SOCS1 silencing on activated macrophage-driven EMT of IECs. RAW264.7 Macrophages were transfected with SOCS1 siRNAs. After 6 h of incubation followed by 24-hour rest, the cells were treated with 100µM UDCA and activated by 100ng/ml of LPS. After 24 h of incubation, the culture supernatant was collected. CT26 cells were treated with macrophage conditioned medium. **(A)** MTT assay was performed to analyze the effect of SOCS1 knockdown in macrophages on activated macrophage culture supernatant-treated CT26 cell proliferation. **(B)** Impact was observed on the colony formation of CT26 cells upon SOCS1 silencing. The regulator effect of UDCA was significantly reduced in the absence of SOCS1. Quantified histogram representation of relative colony number and the absorbance of stain taken up by the cells. **(C)** Representative images from in vitro wound healing assays after 48 h of incubation demonstrated that cell migration significantly reduced on UDCA treatment but was reversed upon SOCS1 silencing. **(D)** Representative images showing the results of trans-well invasion assay of CT26 cells treated with SOCS1 deficient macrophage-conditioned medium. Quantified histogram representation of the relative number of cells per field and the absorbance of stain taken up by the cells. **(E)** Western blot analysis of N-Cadherin, Snail, and Slug protein level expression in SOCS1 deficient-macrophage conditioned media-treated CT26 cells after 48 h of incubation. Densitometry w.r.t β-Actin **F**,** G)** Pro-inflammatory cytokine expression and secretion by BA-treated and SOCS1 knockdown macrophages were quantified by qPCR and ELISA, respectively. Data represented as mean ± SD of at least triplicate experiments. *P < 0.05, **P < 0.01, ***P < 0.001, ****P < 0.0001. Raw images of western blots are provided as supplementary files.

To address the contribution of SOCS1 in UDCA-mediated regulation of EMT, a target-specific siRNA knockdown of SOCS1 was performed. siSOCS1 (QIAGEN FlexiTube siRNA, #1027417) transfected RAW264.7 cells were confirmed for the down regulation of SOCS-1 expression by quantitative real-time PCR **(Supplementary Fig. S2B)**. RAW264.7 cells knockdown for SOCS-1 were pre-treated with UDCA for 1 h and activated by 100ng/ml LPS. After 24 h of incubation, the conditioned media was collected and brought into contact with CT26. The cell proliferation, colony formation, migration, and invasion properties attenuated by UDCA were re-established in SOCS1 knockdown macrophage culture supernatant-treated CT26 cells (Fig. 4A-D). The expression of mesenchymal markers was also enhanced compared to the UDCA-treated cells (Fig. 4E). A similar pattern was observed in the inflammatory cytokine expression at the level of mRNA and protein in macrophages (Fig. 4F, G).

### UDCA inhibits AOM-DSS-induced tumor formation in vivo

**Fig. 5:**
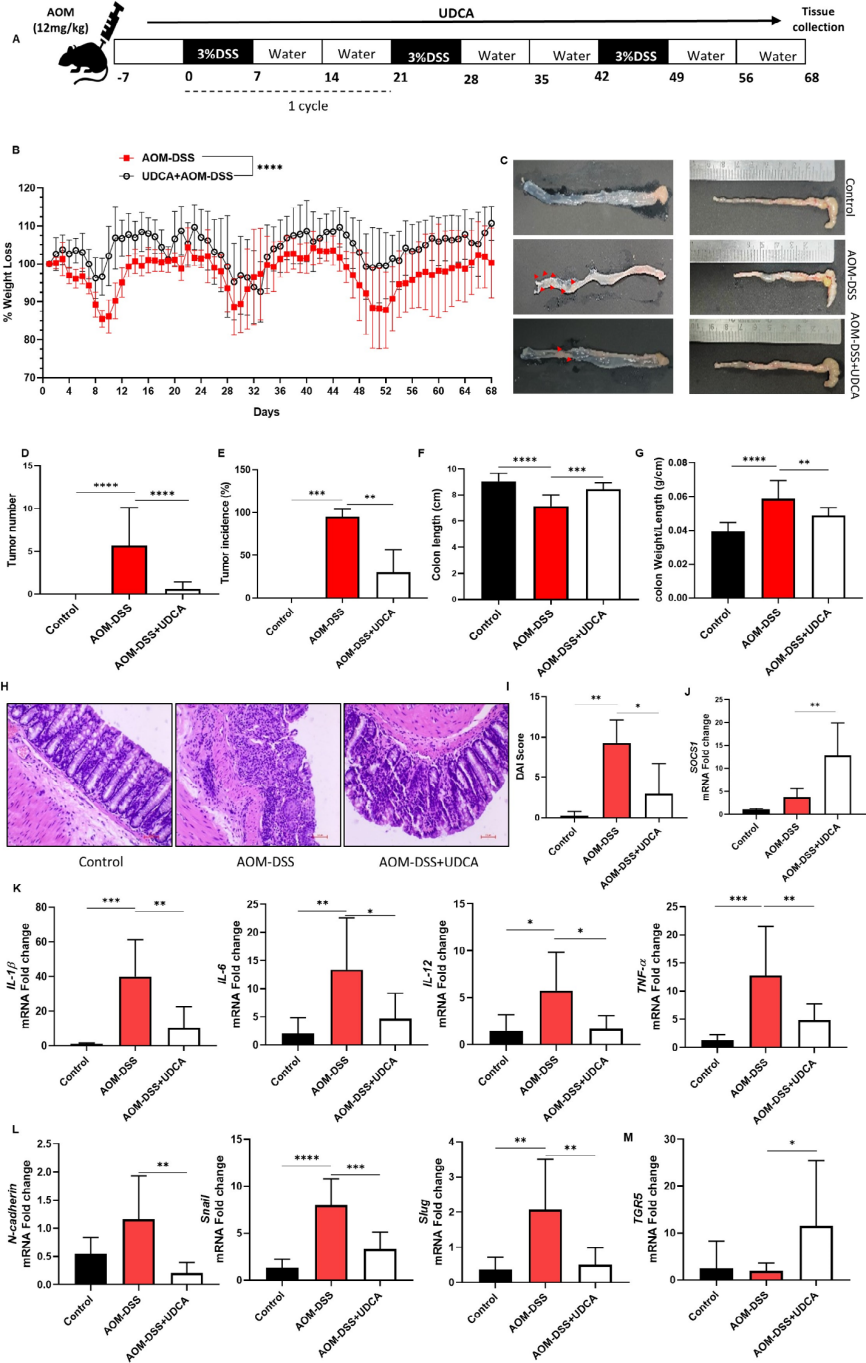
AOM-DSS-induced colorectal cancer model treated with UDCA powdered diet. (**A**) The experimental procedure for developing the AOM-DSS-induced CAC model and 0.2% UDCA administration. (**B**) Representative graph of Percentage of Weight loss 7 days post AOM injection, from the initiation of DSS cycle 1. (**C**) A representative image of the colon highlights the visual difference in colon length and tumor occurrence. (**D**) Representative graph showing the occurrence of colon tumors in the groups (**E**) Tumor incidence % (**F**) Colon length in cm (**G**) Colon weight/length ratio (**H**) H & E Staining of representative histological sections of colons from the groups (200 × magnification). (**I**) DAI Score was calculated based on the colon morphology, mucosal integrity, crypt integrity, and immune cell infiltration. (**J**) mRNA expression of SOCS1 **K**) mRNA expression of pro-inflammatory cytokines like IL- 1β, IL-6, IL-12, and TNF-α **L**) mRNA expression of mesenchymal markers N-Cadherin, Snail, and Slug **M**) mRNA expression of TGR5 receptor in colon tissue samples were quantified, and impact of UDCA was inferred. Data represented as mean ± SD of *n* = 12–13. *P < 0.05, **P < 0.01, ***P < 0.001, ****P < 0.0001.

AOM-DSS model of colitis-associated cancer was induced, as shown in Fig. 5A, and colitis-driven colon cancer disease parameters such as weight loss (Fig. 5B), colon length, tumor incidence, and colon morphology were analyzed **(**Fig. 5C-G**)**. Histological assessment indicated colon inflammation was characterized by loss of mucosal integrity. UDCA treatment reduced mucosal erosion, maintained crypt integrity, and decreased inflammatory cell infiltration (Fig. 5H, I**)**. In consonance with in vitro data, the mRNA level of SOCS1 was also found to be significantly high upon treatment with UDCA (Fig. 5J**)**. Consistent with the morphological and histological improvement, the mRNA expression of pro-inflammatory cytokines IL-1β, IL-6, IL-12, and TNF-α was also decreased in colon tissues from UDCA-treated mice (Fig. 5K). A similar impact was observed in the mRNA expression of mesenchymal markers, indicating UDCA-regulated inflammation-driven EMT (Fig. 5L). Interestingly, the mRNA expression of TGR5 was upregulated in the colon tissue from UDCA-treated mice (Fig. 5M). This increased expression of TGR5 may also have contributed to the ameliorating effect of UDCA reducing inflammation.

These data corroborate the protective role of UDCA by reducing pro-inflammatory gene expression and as well modulating EMT marker expression along with shedding light on the possible involvement of SOCS1 in UDCA-mediated tumor suppression.

## Discussion

One of the mechanisms that can contribute to the development of chronic inflammation to carcinogenesis involves cytokines produced by intestinal immune cells and epithelial cells in response to inflammatory stimuli^37^. The tightly regulated crosstalk between intestinal epithelial cells and the gut immune system ensures the balance between pro-inflammatory signals and anti-inflammatory regulators. Under healthy conditions, this interaction helps to maintain the tolerance towards the gut microbiota^38^. In IBD, the immune homeostasis is disturbed, activating various inflammatory responses and contributing to an increase in inflammatory cytokine expression^19,20^.

This study highlights the therapeutic potential of gut-microbiota-derived host secondary metabolites in regulating inflammation and, thereby, inflammation-driven malignancies. These secondary metabolites are potential immune response modulators in inflammatory diseases^38,39^. Preclinical studies in IBD models and mouse colitis models have progressively demonstrated the anti-inflammatory impact of UDCA as it reduces the secretion and accumulation of pro-inflammatory cytokines, including TNF-α, IL-6, IL-12, and IL-1β, along with lowering the immune cell infiltration into the mucosa^40–42^. The exact mechanism by which UDCA succeeds in finetuning the immune response is still unclear.

In this study, we focused on understanding the role of UDCA in regulating inflammatory responses and contributing to intestinal health and immune homeostasis. Consistent with prior studies, UDCA significantly reduced the expression and secretion of pro-inflammatory cytokines TNF-α, IL-6, IL-12, and IL-1β in LPS-activated macrophages. To explore the involvement of UDCA in the dialogue between activated macrophages and intestinal epithelial cells in an inflamed environment, we treated colon epithelial cancer cell line CT26 with the conditioned medium from activated RAW264.7 macrophages with and without UDCA treatment. This co-culture setup revealed the modulatory effect of UDCA in activated macrophage-driven EMT (Fig. 1).

The inflammatory response of UDCA-treated activated macrophages on CT26 was reversed to a significant extent by siRNA-mediated knockdown of TGR5 receptors on macrophages, indicating the essential role of TGR5 receptors in facilitating the anti-inflammatory role of UDCA (Fig. 2). TGR5 was first described as a BAR in monocytes and macrophages, where it suppressed their function. This function occurs through regulating various signaling pathways, predominantly inhibiting cytokine production^30^.

FXR is known to play a crucial role in colorectal cancer, liver cancer, and cholestatic diseases. However, conflicting reports exist regarding the impact of UDCA on FXR activation. Several reports suggest the antagonist potential of UDCA on FXR activation, whereas on the contrary, some studies classify UDCA as a weak agonist^43–45^. Though UDCA is known to be a weak agonist of TGR5, several studies have explored the ability of UDCA to completely or partly activate TGR5 signaling. Some of these studies include the work by Zhang H.et al., which indicates the anti-cancer potential of UDCA through the TGR5-YAP axis in colorectal cancer, and a study by Zhang, Feng et al. highlighting the anti-inflammatory potential of UDCA in microglia through TGR5 receptor activation^46,47^.

Downstream of TGR5 signaling involves the activation of adenylate cyclase, causing an increase in cAMP, which further triggers the activation of Protein Kinase A (PKA). TGR5 signaling has been reported to inhibit NF-κB activation via the cAMP-PKA pathway in macrophages, also leading to the downregulation of pro-inflammatory cytokines like IL-1β, IL-6, TNF-α, and IL-12p70^48^. Our study suggests the involvement of the TGR5 receptor in UDCA-mediated regulation of inflammation and inflammation-driven EMT.

Cytokines are key modulators of immune cells, mainly by modifying the transcriptional profile to upregulate or downregulate particular genes. Cytokine-activated signaling often leads to the induction of SOCS proteins and acts as a classical negative feedback loop to inhibit cytokine signal transduction^49^. SOCS1 is known to play a protective role from LPS responses. A study with SOCS1 deficient mice exhibited high sensitivity to LPS. SOCS1 is also known to regulate the uptake of LPS in mouse hepatocytes, preventing sepsis. SOCS3 also plays an important role in regulating LPS-triggered inflammation by targeting multiple pro-inflammatory cytokines. Reports suggest that the depletion of SOCS3 in macrophages positively regulates TLR4 signaling. Also, SOCS3 modulates bone-associated inflammation in osteoblasts by inhibiting LPS-induced IL-6 and blocking CAAT/enhancer-binding proteins (C/EBPβ)^50^.

SOCS1 instigates several mechanisms for the suppression of cytokine production and is known to play a crucial role in the negative regulation of the TLR pathway during the development of IBD, making it a potential target for the treatment of inflammatory diseases involving hyper-cytokine signaling^51,52^. One approach is the over-expression of SOCS1, finetuning the feedback loop and keeping the cytokine expression in check. In this study, we could observe a significant increase in the expression of SOCS1, SOCS3, and CISH isoforms in macrophages treated with UDCA (Fig. 3), indicating that the anti-inflammatory potential of UDCA can be attributed to its ability to upregulate the expression of negative regulators of cytokine.

UDCA significantly upregulated SOCS1 among the isoforms quantified in macrophages. To further understand the SOCS1 dependency of UDCA-mediated inhibition of EMT, SOCS1-specific siRNA-mediated knockdown was employed in macrophages. These macrophages were further treated with UDCA and subsequently activated by LPS. After 24 h of incubation, the supernatant was collected. CT26 cells treated with only LPS-conditioned medium showed increased proliferation, migration, and colony-forming ability. This outcome was significantly reduced to a great extent in the presence of UDCA. Interestingly, the transient knockdown of SOCS1 in macrophages diminished the regulatory effect of UDCA (Fig. 4), suggesting that UDCA depends on SOCS1 for its immuno-modulatory activity.

**Fig. 6:**
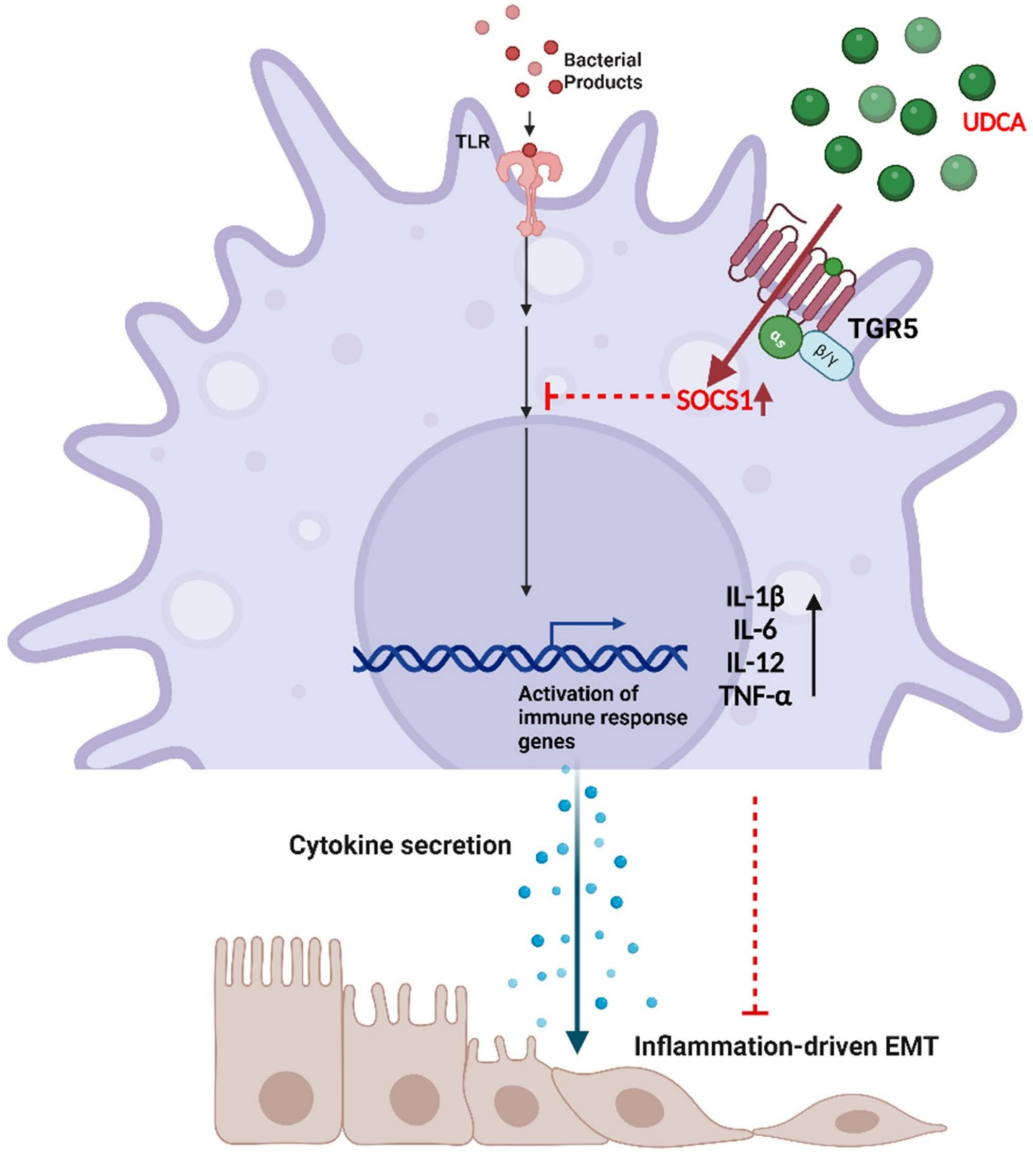
Graphical abstract. Schematic representation of UDCA ameliorating activated macrophage-driven EMT involving TGR5 and SOCS1 expression. (Created using BioRender.Com).

BA-induced immune modulation has been shown to mediate the prognosis of colitis^53^. The evident role of UDCA in suppressing inflammatory response makes it a standard therapeutic strategy for inflammatory diseases^40^. Similarly, the present study demonstrated that UDCA ameliorated chronic inflammation-driven colitis and tumorigenesis in a mouse model by suppressing intestinal inflammation, further emphasizing the clinical significance of UDCA in colitis and CAC. In support of previous observations, we could observe a heightened expression of SOCS1 and TGR5 and a significant reduction in pro-inflammatory cytokines in colon tissues obtained from UDCA-fed mice. However, no significant upregulation of other BARs and SOCS isoforms was observed (**Supplementary Fig. S3)**. It was also accompanied by the expected decline in tumor formation, moderation of immune cell intrusion, maintenance of mucosal integrity, and healthier colon morphology (Fig. 5). These observations collectively highlight the importance of UDCA and SOCS1 in intestinal immune modulation and the possible involvement of SOCS1 in modulating colorectal cancer via the TGR5 receptor (Fig. 6).

However, conflicting results from recent studies, including experimental and clinical samples, indicate the ambiguous potential of SOCS1 as either a tumor suppressor or a tumor promoter. This suggests the need to explore deeper into the cell-specific dual role of SOCS1 as well as its other isoforms. Our study provides insight into the possible role of gut microbiota-derived secondary metabolites, such as UDCA, as an anti-cancer therapeutics that can act as a molecular switch dictating SOCS1 functionality towards tumor suppression by regulating inflammation and, in the long run, inflammation-driven colon cancer.

## Materials and methods

### Cells and cell culture

A mouse macrophage cell line RAW 264.7 and colon cancer epithelial cell line CT26 were purchased from NCCS-Pune, India. The cells were cultured in complete DMEM (Thermo Scientific, Gibco, #12100061) supplemented with 10% heat-inactivated FBS (Thermo Scientific, Gibco Brazil, #10270106) and 1X PenStrep (Thermo Scientific, #15140122) at 37˚C and 5% CO_2_. Thioglycolate-elicited mouse peritoneal macrophages were obtained as described. Briefly, 2X Thioglycolate solution was injected into the mice’s peritoneal cavity. After four days, the cells were harvested from the peritoneal cavity in ice-cold 1X PBS, counted, and seeded as required. Once cultured in complete DMEM at 37˚C and 5% CO_2_, the peritoneal macrophages become adherent, allowing their separation from other types of cells isolated from the peritoneal cavity. All the mice experiments were carried out after obtaining approval from the Institutional Animals Ethics Committee (IAEC), and the protocol adhered to was granted approval by national guidelines of the Committee for Control and Supervision of Experiments on Animals (CCSEA) (formerly CPCSEA), Government of India (IAEC No: BITS-Hyd/IAEC/FA/2018/1). The study reported is in accordance with ARRIVE guidelines. Additionally, all the experiments were performed as per the approval by Institutional Biosafety Committee (IBSC) Guidelines-BITS Pilani, Hyderabad Campus.

### Bile acid treatment and LPS-mediated activation of mouse macrophages

Isolated macrophages were treated with a non-toxic concentration (100µM) of BAs of interest. The treatment was followed by activating macrophages upon exposure to 100 ng/ml LPS (Sigma, #L4391) and incubating for 24 h. The conditioned culture supernatant was then collected to study its impact on regulating inflammation-driven EMT of intestinal epithelial cells (CT26).

### Transient knockdown of bile acid receptors

Independent siRNAs targeting BARs were purchased from Ambion, life technologies, and reverse transfection was performed using Lipofectamine RNAiMAX (Invitrogen, #13778-100) as per the manufacturer’s protocol. Briefly, RAW 264.7 cells were seeded in a 12-well plate in complete DMEM at a seeding density of 0.1 × 10^6^ cells/well and allowed to adhere overnight. 5pmol of target-specific siRNAs/NT siRNA and Lipofectamine was thoroughly mixed in a serum-free medium and incubated at room temperature for 20–30 min. After optimization, the volume for each reaction was made up to 1 ml with serum-free media, and cells were treated. The cells were incubated for 6 hours along with siRNA-lipid complex. The media was replaced with complete DMEM, and cells were allowed to recover for 24 h. The treatment with 100µM BAs was followed by the activation of macrophages by 100ng/ml of LPS. The supernatant was collected after 24 h of incubation for further experiments. All the siRNA sequences used in the study were listed in Supplementary Table S1.

### Cell proliferation assay

For cell viability and proliferation, MTT Assay was performed. CT26 cells with a density of 2000 cells/100µl media were seeded in a 96-well plate and, once adhered completely, treated with the conditioned media from BA-treated activated macrophages. The conditioned media was added in a 1:4 ratio and was followed by cell viability analysis using MTT reagent as per protocol, and absorbance was taken at 0 h, 24 h, 48 h, and 72 h. Briefly, 10 µl of MTT solution (5 mg/ml) was added to each well and incubated for 1–4 h at 37 ˚C. The formazan crystals formed were dissolved in 200 µl of DMSO, and absorbance was recorded at 570 nm.

### Colony forming assay

CT26 cells were seeded in a 6-well culture plate at 300 cells/well density. After cells adhered completely, they were incubated in the conditioned medium in a 1:1 ratio for 7–9 days. The colonies formed were fixed with a 3:1 ratio of methanol and glacial acetic acid for 10 min. The wells were then washed with 1X PBS, and fixated cells were stained with 0.5% v/v crystal violet solution for 10 min. The excess stain was washed off in running water, and images were taken after drying. Colonies were counted visually or using ImageJ software. The stain taken up by the colonies was dissolved in 10% SDS, and absorbance was taken at 540 nm.

### Wound healing assay

0.2 × 10^6^ CT26 cells were seeded in a 12-well tissue culture plate and incubated till a uniform monolayer was formed. Once confluent, the cell layer was scraped in a straight line using a 200 µl pipette tip, keeping the tip perpendicular to the bottom of the well. After the scratch, a gentle wash was given to remove detached cells. The cells were incubated with a 1:1 ratio of the conditioned medium from treated macrophages, and images were taken under a microscope on 4X magnification at 0 h, 24 h, and 48 h. The percentage of wound closure was calculated to evaluate the antiproliferative effect of the BAs-treated macrophage culture supernatant.

### Invasion assay

The invasion ability of the cells upon exposure to activated macrophage-conditioned media and the regulatory effect of UDCA was tested by transwell assay. CT26 cells were seeded at a seeding density of 0.01 × 10^6^ cells/well into the upper chamber of transwell inserts (Corning, #3422) precoated with 0.2% Gelatin (Sigma, #G2500) in a 1:1 ratio of conditioned medium and serum-free media. The lower chamber was loaded with complete DMEM with 10% FBS, sufficient to touch the bottom of the inserts. After 48 h of incubation, the inserts were gently washed in 1X PBS, and the cells remaining in the upper chamber were removed with a cotton swab. The cells that invaded the other side of the insert membrane were fixed with 4% Paraformaldehyde for 15 min, followed by permeabilization with 100% methanol for 20–30 min. The cells were stained with crystal violet for 20 min, washed, and after proper drying, images were taken at five different fields at 10X objective magnification. The average number of cells per field was recorded. The stains taken up by the cells were dissolved in 10% SDS, and absorbance was taken at 540 nm.

### Western blotting

0.1 × 10^6^ cells/well of CT26 were seeded in a 12-well cell culture plate and allowed to adhere properly. The cells were then incubated with a 1:1 ratio of the conditioned medium for 48 h. The cells were pelleted in 1X PBS and lysed using ice-cold RIPA buffer consisting of 50 mM Tris-HCl (pH 7.4), 0.25% sodium deoxycholate, 1% NP-40, 1mM EDTA, 150 mM NaCl, 1 µg/ml of aprotinin, 1 µg/ml of pepstatin, 1mM PMSF, and 1mM Na3VO4 on ice for 30 min. The whole cell lysate was centrifuged at 12,000 rpm for 15 min at 4˚C, and the supernatant was collected. The protein concentration of the lysate was quantified using the Bradford assay, and an equal concentration of sample proteins was subjected to SDS-PAGE and transferred onto 0.45 μm PVDF membrane (Millipore, Immobilon #IPVH00010) by using the semidry Western Blotting method. The non-specific binding was blocked with 5% skimmed milk in 1X TBST for 1 h, and after thorough washing, probed with primary antibodies of N-CADHERIN (CST, #13116S), SNAIL (CST, #5879S), SLUG (CST, #9585S), and β-ACTIN (Sigma Aldrich, #A3854) overnight at 4 ˚C and HRP conjugated anti-rabbit IgG secondary antibody (Jackson Immuno Research Laboratories, #111-035003) for 4–5 h in 4 ˚C. After thorough washing the protein signals were visualized using enhanced chemiluminescence (ECL) kit (Bio-Rad, #1705061).

### RT-qPCR

The treated cells were suspended in TRIzol (Thermo Scientific, #15596026) to lyse the cells. This was followed by adding chloroform and pulse vortexing to obtain a homogenous mixture without touching the lid. The tubes were centrifuged at 12,000 rpm for 45 min, 4℃ for phase separation. The aqueous phase was carefully collected into fresh nuclease-free tubes, and equal volumes of isopropanol were added and incubated at -80℃ for 2 h for RNA to precipitate. After proper thawing on ice, centrifuge at 12,000 rpm for 45 min at 4℃, and the pellet was washed with 70% ethanol twice by centrifuging at 12,000 rpm for 10 min at 4℃. The pellet was air-dried and re-suspended in nuclease-free water. The integrity of RNA was determined by performing a 2% Agarose gel electrophoresis, and the purity and amount of RNA were determined by NanoDrop (Thermo Scientific NanoDrop One^C^).

Complementary DNA (cDNA) was synthesized from 1 µg of total RNA from each sample using PrimeScript first Strand DNA synthesis kit (TaKaRa Bio, #6110A) The expressions of specific genes were quantified using gene-specific primers mixed with the TB Green remix Taq II (TaKaRa Bio, #RR820A; under the following conditions: Initial Denaturation 95℃ for 30 s (Ramp Rate 4.4℃/sec); 1 cycle PCR Analysis Mode: Quantification 95℃for 5 s (Ramp Rate 4.4℃/sec), 60℃ for 30 s (Ramp Rate 2.2℃/sec, Acquisition Mode: Single) 40 cycles; Melting Analysis Mode: Melting Curves 95℃ for 5 s (Ramp Rate 4.4℃/sec), 60℃ for 1 min (Ramp Rate 2.2℃/sec) and 95℃ (Ramp Rate 0.11℃/sec, Acquisition Mode: Continuous, Acquisitions: 5 per ℃) 1 cycle; Cooling 50℃ 30 s (Ramp Rate 2.2℃/sec) 1 cycle). The gene expression of interest was normalized using β-Actin, 2 ^–(ΔΔCT)^. All the primers sequences used in the study were listed in Supplementary Table S1.

### ELISA for cytokine expression

RAW264.7 cells were seeded in a 12-well plate at a seeding density of 0.1 × 10^6^ cells/ well. Following adhesion, the cells were pretreated with UDCA for 1 h and activated by 100ng/ml LPS. After 24 h of incubation, the culture medium was collected, and pro-inflammatory cytokines IL-1β (Invitrogen, Thermo Fisher Scientific, #88-7013), IL-6 (PEPROTECH, #900-TM50), IL-12(PEPROTECH, #900-TM97), and TNF-α (PEPROTECH, #900-TM54) in the supernatant were quantified by ELISA according to the manufacturer’s instruction. The absorbance was taken at 450 nm using a microtitre plate reader, and the cytokine secretion level was calculated based on the standard curve plotted.

### Immunofluorescence

Immunostaining was performed to evaluate the expression of negative regulators of cytokine upon UDCA treatment in macrophages. 0.05 × 10^6^ Raw246.7 cells were seeded onto the coverslip and allowed to adhere overnight. The cells were transfected with TGR5 siRNA using Lipofectamine RNAiMAX in serum-free media for 6 hours and were followed by 24-hour rest in complete DMEM. The cells were treated with UDCA and incubated for 24 h. The cells were fixed with 3.7% Paraformaldehyde (PFA) for 15 min at room temperature, after which they were probed with a primary antibody specific to SOCS1 (CST, #3950T) overnight at 4˚C, followed by a fluorescent-tagged secondary antibody (CST, #4412) in the dark for 1 h in room temperature. 10 µg/ml of DAPI was used to stain the nucleus. After thorough washing with 1XPBS to remove the excessive stain, the coverslips were mounted onto glass slides and observed under a confocal microscope at 63X objective magnification. MFI graphs were plotted with the help of ImageJ.

### Animal model

8–12 weeks-old female C57BL/6 mice were purchased from ICMR-National Animal Resource Facility for Biomedical Research (NARFBR). All the protocols were adhered as approved (IAEC No: BITS-Hyd/IAEC/FA/2018/1). Mice were maintained in a specific pathogen-free animal facility under a standard 12:12-h light/dark cycle and were fed standard rodent chow and water ad libitum. The mice were accurately weighed and divided into the (1) control group, (2) AOM-DSS group, and (3) AOM-DSS + UDCA group. In the AOM-DSS treated groups (2 and 3), the mice were injected intraperitoneally with AOM (Sigma-Aldrich, #A5486) at a dosage of 12 mg/kg body weight in combination with three cycles of 3% DSS (MP Biomedicals, colitis grade (36,000–50,000)-25 g, #160110) administered via drinking water for seven days followed by regular water for 14 days. The control and AOM-DSS groups were fed a regular diet [1 and 2], whereas the AOM-DSS + UDCA group^3^ was fed a diet containing 0.2% UDCA throughout the experiment. Weights were recorded regularly, and all the mice were sacrificed at the end of the third cycle. At the end of the experiment, mice were euthanised following approved cervical dislocation method. Colon was obtained, and the length and weight were calculated. Tumor numbers were noted, and colon tissues were collected in Trizol for qRT-PCR analysis and 10% Formalin. Formalin-fixed tissues are embedded into paraffin blocks and sectioned for histological examination.

### Statistical analysis

The graphical data are expressed as mean ± SD of 3 or more independent experiment sets. Statistical tests were performed using GraphPad Prism Software version 8.0.2. Comparisons of the observed data were analyzed using Student *t* test distribution and one-way/two-way ANOVA where ever applicable.

## Electronic supplementary material

Below is the link to the electronic supplementary material.

Supplementary Material 1

## Data Availability

The publicly available datasets have been analyzed and data obtained is shown in the supplementary figure. The name of the repository and accession number that have been accessed and analyzed to derive a conclusion can be found in the following: https://www.ncbi.nlm.nih.gov/, GSE68838. TCGA Analysis of DNA Methylation for COAD using Illumina Infinium HumanMethylation450 platform.
