# Author Correction: UDCA ameliorates inflammation driven EMT by inducing TGR5 dependent SOCS1 expression in mouse macrophages

**DOI:** 10.1038/s41598-024-80577-x

**Published:** 2024-11-26

**Authors:** Ashna Fathima, Trinath Jamma

**Affiliations:** Cell Signaling Laboratory, Department of Biological Sciences, Birla Institute of Technology, and Science-PilaniHyderabad Campus, Jawahar Nagar, Shameerpet Mandal, Hyderabad, 500078 Telangana India

Correction to: *Scientific Reports* 10.1038/s41598-024-75516-9, published online 16 October 2024

The original version of this Article contained errors in Figure 5, where due to corruption in the files during revision, several errors were implemented in the statistics shown in the figure. Additionally, a mistake was made in Figure 5A, in which the time period for Tissue Collection was erroneously mentioned as 63 instead of 68.

The original Figure 5 and accompanying legend appear below.

**Fig. 5:**
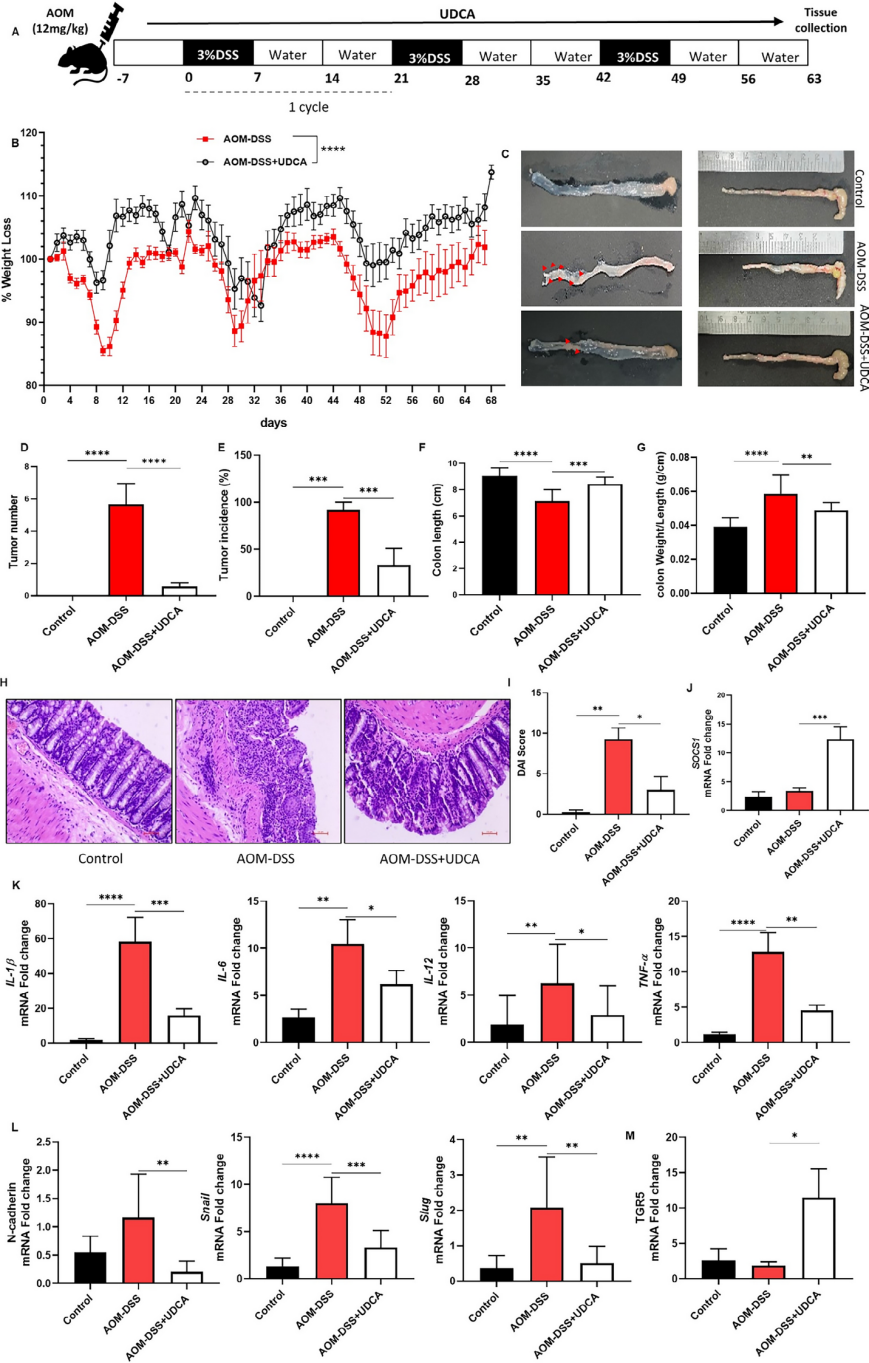
AOM-DSS-induced colorectal cancer model treated with UDCA powdered diet. **(A)** The experimental procedure for developing the AOM-DSS-induced CAC model and 0.2% UDCA administration. **(B)** Representative graph of Percentage of Weight loss 7 days post AOM injection, from the initiation of DSS cycle 1. **(C)** A representative image of the colon highlights the visual difference in colon length and tumor occurrence. **(D)** Representative graph showing the occurrence of colon tumors in the groups **(E)** Tumor incidence % **(F)** Colon length in cm **(G)** Colon weight/length ratio **(H)** H & E Staining of representative histological sections of colons from the groups (200× magnification). **(I)** DAI Score was calculated based on the colon morphology, mucosal integrity, crypt integrity, and immune cell infiltration. **(J)** mRNA expression of SOCS1 **(K)** mRNA expression of pro-inflammatory cytokines like IL- 1β, IL-6, IL-12, and TNF-α **(L)** mRNA expression of mesenchymal markers N-Cadherin, Snail, and Slug **(M)** mRNA expression of TGR5 receptor in colon tissue samples were quantified, and impact of UDCA was inferred. Data represented as mean ± SD of *n* = 12–13. *P < 0.05, **P < 0.01, ***P < 0.001, ****P < 0.0001.

The original Article has been corrected.

