# Supplementary material for: UDCA ameliorates inflammation driven EMT by inducing TGR5 dependent SOCS1 expression in mouse macrophages
Source: Sci Rep. 2024 Oct 16;14:24285. doi: 10.1038/s41598-024-75516-9 (PMC11484976; doi:10.1038/s41598-024-75516-9)
Supplement: Supplementary file 1 — Supplementary Material 1 [file 41598_2024_75516_MOESM1_ESM.pdf]

UDCA ameliorates inflammation driven EMT by inducing TGR5 dependent SOCS1 expression in mouse macrophages

Ashna Fathima[1], Trinath Jamma[1*]

[1]Cell Signaling Laboratory, Department of Biological Sciences, Birla Institute of Technology and Science-Pilani, Hyderabad Campus, Jawahar Nagar, Shameerpet Mandal, Hyderabad, Telangana State 500078, India

*Correspondence author: trinath@hyderabad.bits-pilani.ac.in; karunya.friend@gmail.com

Supplementary Table S1: qPCR primer sequences and siRNA duplex sequences utilized.

qPCR Primer Sequences-

	**Forward primer**	**Reverse primer**
**mSOCS1**	CTGCGGCTTCTATTGGGGAC	AAAAGGCAGTCGAAGGTCTCG
**mSOCS2**	GATAGGACGAGTTCCCCACA	TGTGCAAGGATAAACGGACA
**mSOCS3**	ATGGTCACCCACAGCAAGTTT	TCCAGTAGAATCCGCTCTCCT
**mCIS**	GGGTGCTGTCTCGAACTAGG	CCCAGAGGAAGTGACAGAGG
**mTGR5**	CTGTGTGAGATCCGCCGAC	CGACGCTCATAGGCCAAGA
**mβ-Actin**	GGCTGTATTCCCCTCCATCG	CCAGTTGGTAACAATGCCATGT
**mIL-1β**	GCACTACAGGCTCCGAGATGAAC	TTGTCGTTGCTTGGTTCTCCTTGT
**mIL-6**	CTTCTTGGGACTGATGCTGGTG	CAGGATTTCCCAGAGAACATGTG
**mIL-12**	TGGTTTGCCATCGTTTTGCTG	ACAGGTGAGGTTCACTGTTTCT
**mTNF-α**	CCTGTAGCCCACGTCGTAG	GGGAGTAGACAAGGTACAACCC
**mSlug**	TGGTCAAGAAACATTTCAACGCC	GGTGAGGATCTCTGGTTTTGGTA
**mSnail**	CACACGCTGCCTTGTGTCT	GGTCAGCAAAAGCACGGTT
**mN-Cadherin**	AGCGCAGTCTTACCGAAGG	TCGCTGCTTTCATACTGAACTTT

siRNA duplex sequences-

	**Sense**	**Antisense**
**NT siRNA**	UUCUCCGAACGUGUCACGUdTdT	ACGUGACACGUUCGGAGAAdTdT
**mTGR5 siRNA**	GGAACUCUGUUAUCGCUCAtt	UGAGCGAUAACAGAGUUCCag
**mFXR siRNA**	GAAAUCCAGUGUAAAUCUAtt	UAGAUUUACACUGGAUUUCag
**mPXR siRNA**	CCAUCAACGUAGAGGAGGAtt	UCCUCCUCUACGUUGAUGGgc
**mVDR siRNA**	CCAAUUCGUGCAGACGUAAtt	UUACGUCUGCACGAAUUGGag

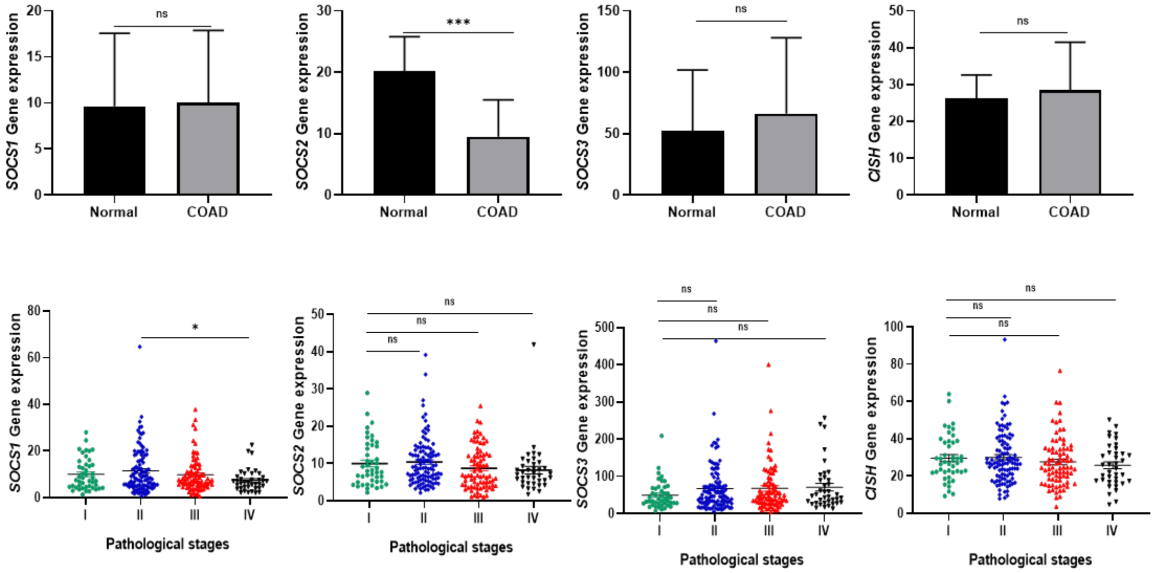

Supplementary Fig. S1: TCGA data analysis for SOCS isoforms in Normal vs COAD patients. Relative gene expression of SOCS isoforms in colonic biopsies data set available from TCGA (oncodb.org). Data represented as mean ± SD. **P<0.01, ***P<0.001, ****P<0.0001 vs normal or COAD patients stage 1.

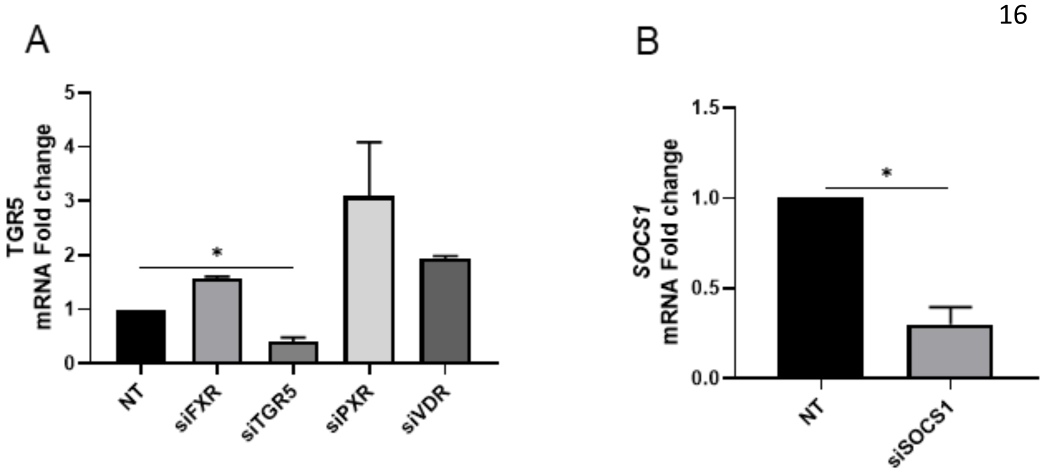

Supplementary Fig. S2: Quantification of TGR5. Relative mRNA expression of A) BARs upon siRNA-mediated TGR5 silencing and B) SOCS1 in siRNA-mediated SOCS1 silencing in RAW264.7 cells. Data represented as mean ± SD. *P<0.05

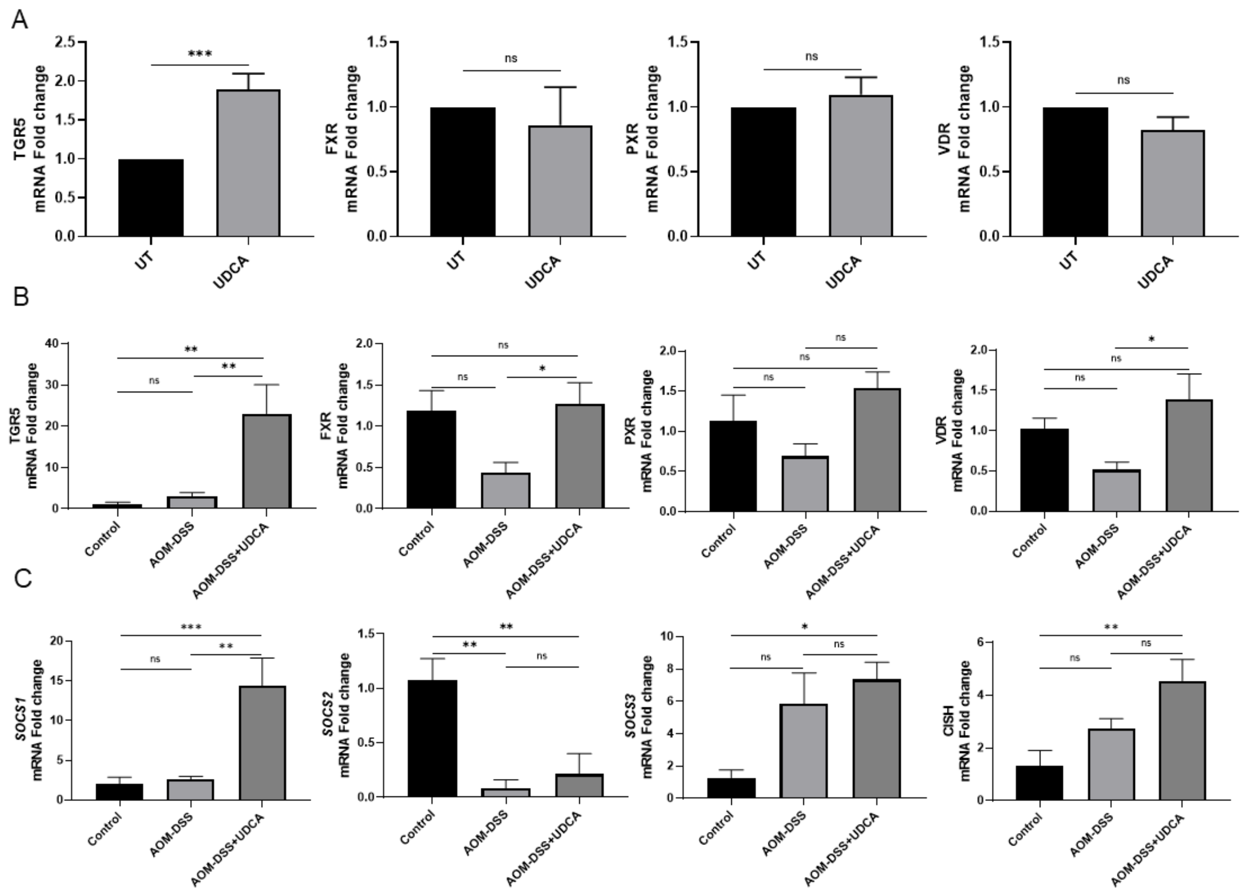

Supplementary Fig. S3: qRT-PCR analysis

Relative mRNA expression of A) BARs in UDCA-treated RAW264.7 cells in vitro B) BARs in UDCA-fed AOM-DSS mice model in vivo C) SOCS isoforms in UDCA-fed AOM-DSS mice model in vivo. Data represented as mean ± SD. **P<0.01, ***P<0.001

Raw western blot images :

Figure 1E:

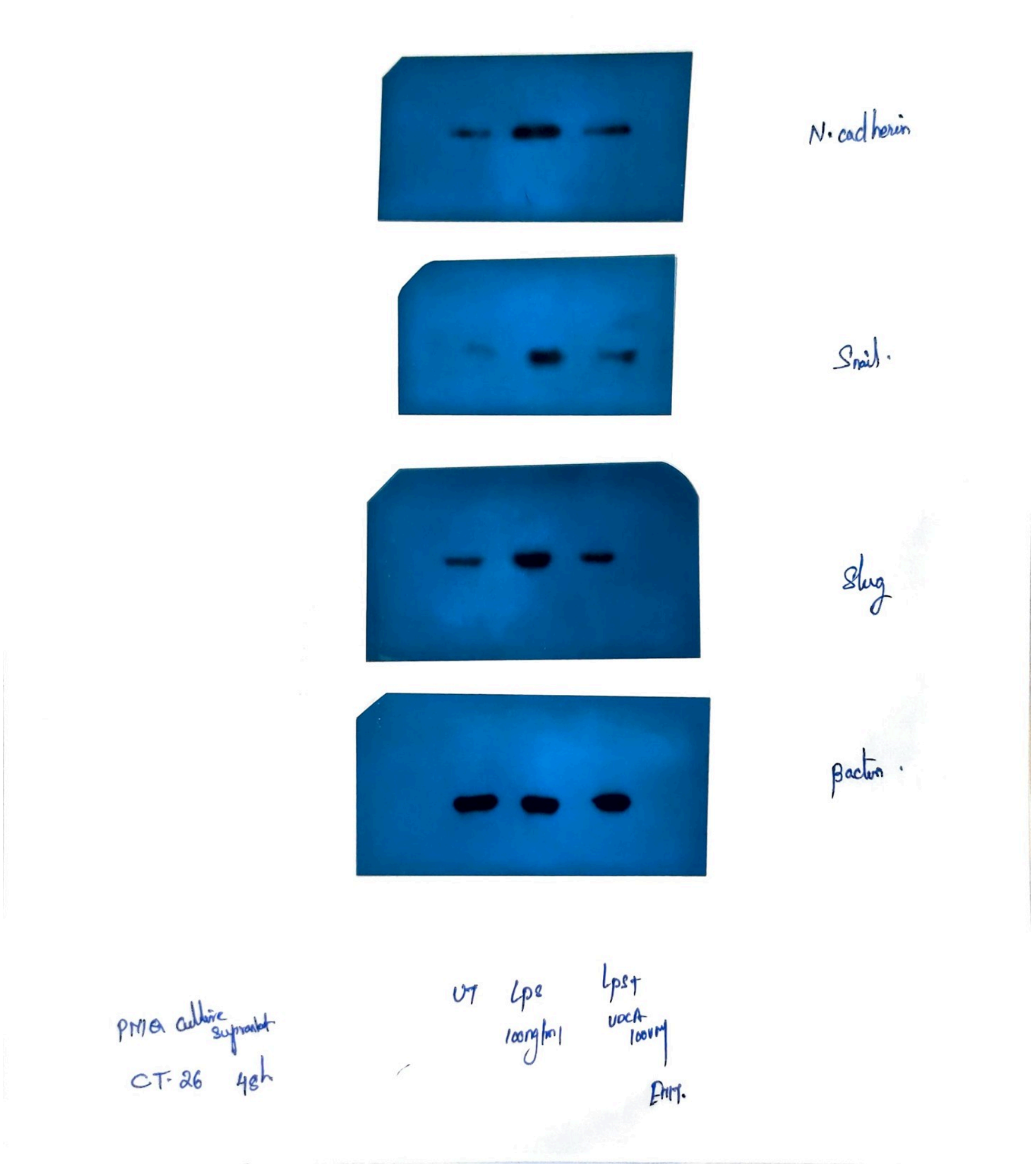

Figure 2E:

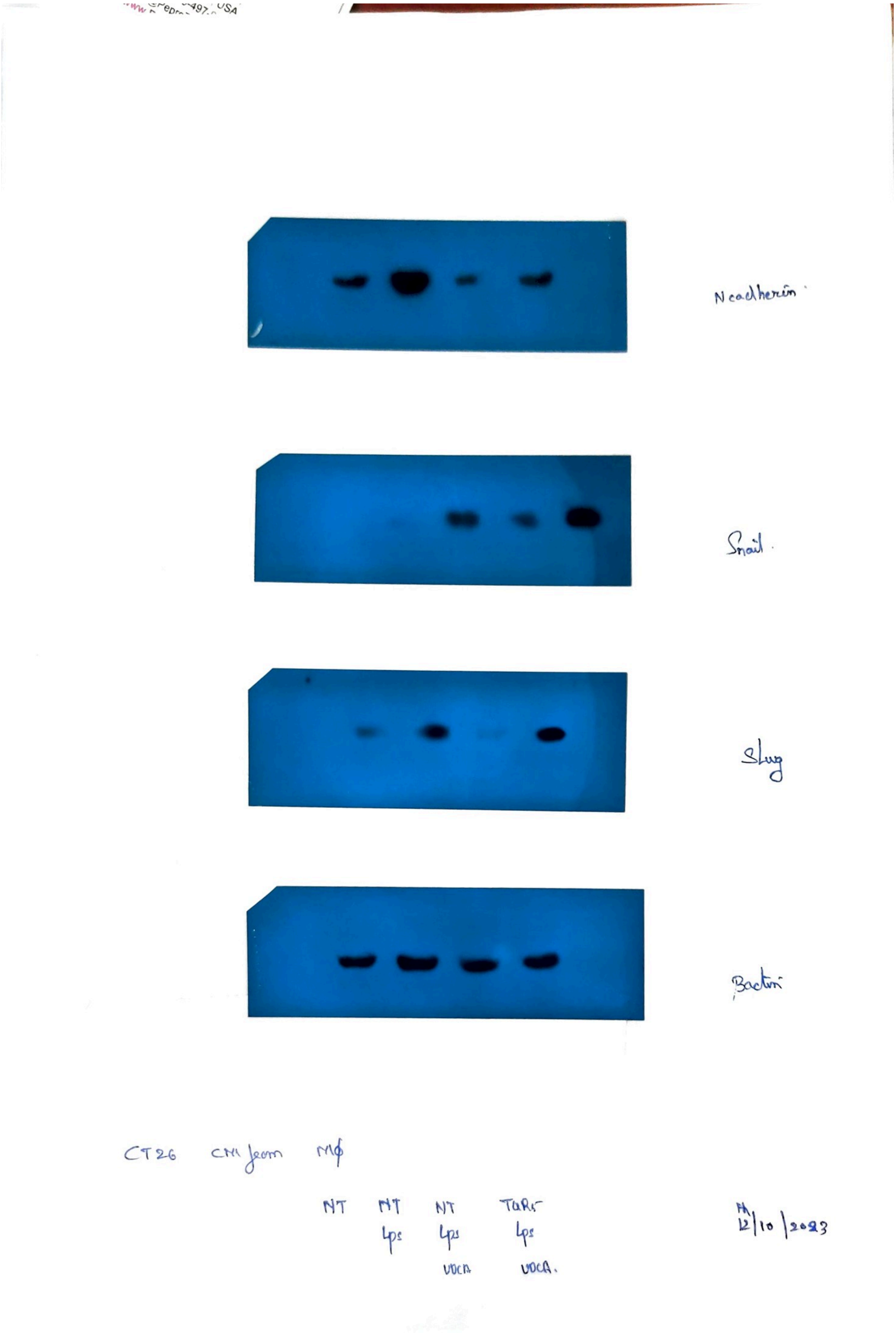

Figure 4E:

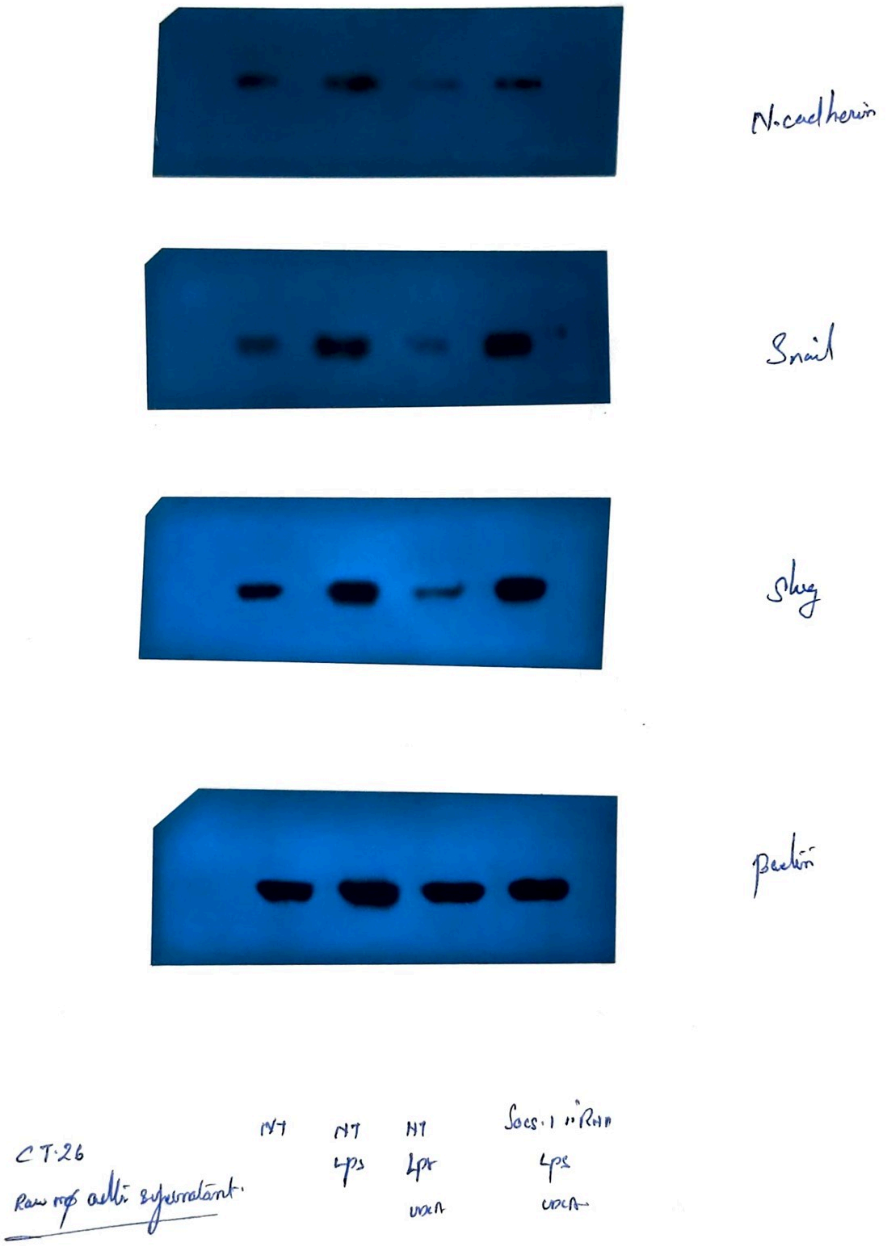
N-cadherin
Snail
Slug
β-actin
CT-26
Raw mφ cells supernatant.
NT
NT
Lps
NT
Lps
UDCA
Socs-1 siRNA
Lps
UDCA
